# Supplementary material for: Gut microbial features can predict host phenotype response to protein deficiency
Source: Physiol Rep. 2018 Dec 4;6(23):e13932. doi: 10.14814/phy2.13932 (PMC6280014; doi:10.14814/phy2.13932)
Supplement: Supplementary file 1 — Table S1. Analyses of Composition of Microbiome (ANCOM) between the four strains of mice i.e. CD1, C57Bl6/J, FVB, NIH‐Swiss on normal chow (NC) diet. Table S2. Analyses of Composition of Microbiome (ANCOM) between the C57Bl6/J mice on protein deficient (PD) and Normal Chow diet (NC). Table S3. Analyses of Composition of Microbiome (ANCOM) between the CD1 mice on protein deficient (PD) and Normal Chow diet (NC). Table S4. Analyses of Composition of Microbiome (ANCOM) between the FVB mice on protein deficient (PD) and Normal Chow diet (NC). Table S5. Analyses of Composition of Microbiome (ANCOM) between the NIH‐Swiss mice on protein deficient (PD) and Normal Chow diet (NC). Table S6. Analyses of Composition of Microbiome (ANCOM) between the four mice strains ,that is, C57Bl6/J, CD1, GVB, NIH‐Swiss on protein deficient (PD). Table S7. Analyses of Composition of Microbiome (ANCOM) between the C57Bl6/J and CD1 mice on protein deficient (PD). Table S8. Rodent Diet Composition. Table S9. Overall microbial community profiles in the three strain of mice Figure S1. Differentially abundant functional enzymes between C57Bl6/J mice on PD and NC diets. All the enzymes were identified after applying Benjamini–Hochberg (BH) False Discovery Rate (FDR) correction to P‐values (<0.05). Figure S2. Differentially abundant functional enzymes between CD‐1 mice on PD and NC diets. All the enzymes were identified after applying Benjamini–Hochberg (BH) False Discovery Rate (FDR) correction to P‐values (<0.05). Figure S3. Differentially abundant functional enzymes between FVB mice on PD and NC diets. All the enzymes were identified after applying Benjamini–Hochberg (BH) False Discovery Rate (FDR) correction to P‐values (<0.05). Figure S4. Differentially abundant functional enzymes between NIH‐Swiss mice on PD and NC diets. All the enzymes were identified after applying Benjamini–Hochberg (BH) False Discovery Rate (FDR) correction to P‐values (<0.05). Figure S5. List of differentially abundant [file PHY2-6-e13932-s001.pdf]

Table S1: Analyses of Composition of Microbiome (ANCOM) between the four strains of mice i.e. CD1, C57Bl6/J, FVB, NIH-Swiss on normal chow (NC) diet. All the Exact Sequence Variants (ESVs) were identified after applying Benjamini-Hochberg (BH) False Discovery Rate (FDR) correction to P-values (< 0.05).

ESVs	C57BL/6_NC MRF (%)	CD-1_NC MRF (%)	FVB_NC MRF (%)	NIH_NC MRF (%)	p-values (corrected)	Effect size
p__Bacteroidetes_c__Bacteroidia_o__Bacteroidales_f__[Odoribacteraceae]_g__Odoribacter_s__	0	0.009649078	9.054081445	2.339399793	1.64E-05	0.871099173
p__Bacteroidetes_c__Bacteroidia_o__Bacteroidales_f__[Paraprevotellaceae]_g__[Prevotella]_s__	0	0	0.995719462	0.002257744	1.32E-05	0.600330676
p__Bacteroidetes_c__Bacteroidia_o__Bacteroidales_f___g___s__	0	0.052816007	5.193274372	2.606381017	1.45E-05	0.835264733
p__Bacteroidetes_c__Bacteroidia_o__Bacteroidales_f__Bacteroidaceae_g__Bacteroides_s__	2.775805537	11.79927773	2.875019	3.294205303	0.006858033	0.570091943
p__Bacteroidetes_c__Bacteroidia_o__Bacteroidales_f__Porphyromonadaceae_g__Parabacteroides_s__distasonis	0.001270245	0	0.005347755	0.045795823	0.001353562	0.618464901
p__Bacteroidetes_c__Bacteroidia_o__Bacteroidales_f__Rikenellaceae_g__AF12_s__	0	0.003047077	0.71224852	0.47852924	2.12E-05	0.809342255
p__Firmicutes_c__Bacilli_o__Turicibacterales_f__Turicibacteraceae_g__Turicibacter_s__	3.702252816	0.047786737	0.060790304	1.730962078	0.001150899	0.624447502
p__Proteobacteria_c__Betaproteobacteria_o__Burkholderiales_f__Alcaligenaceae_g__Sutterella_s__	1.224363878	0.07831591	0	0	5.00E-06	0.632381103

* MRF = mean relative frequency

Table S2: Analyses of Composition of Microbiome (ANCOM) between the C57Bl6/J mice on protein deficient (PD) and Normal Chow diet (NC). All the Exact Sequence Variants (ESVs) were identified after applying Benjamini-Hochberg (BH) False Discovery Rate (FDR) correction to P-values (< 0.05).

ESVs	C57BL/6_PD MRF (%)	C57BL/6_NC MRF (%)	p-values (corrected)	DBM
p__Bacteroidetes_c__Bacteroidia_o__Bacteroidales_f__Bacteroidaceae_g__Bacteroides_s__	7.280470717	2.775805537	0.023976024	4.50466518
p__Bacteroidetes_c__Bacteroidia_o__Bacteroidales_f__S24-7_g___s__	19.0509963	48.04930871	0.000999001	-28.99831241
p__Deferribacteres_c__Deferribacteres_o__Deferribacterales_f__Deferribacteraceae_g__Mucispirillum_s__schaedleri	1.400407562	0.000648298	0.014985015	1.399759264
p__Firmicutes_c__Bacilli_o__Turicibacterales_f__Turicibacteraceae_g__Turicibacter_s__	1.039937005	3.702252816	0.002997003	-2.66231581
p__Firmicutes_c__Clostridia_o__Clostridiales_f__Lachnospiraceae_g__Coprococcus_s__	1.734292665	0.221109571	0.001998002	1.513183094
p__Firmicutes_c__Clostridia_o__Clostridiales_f__Ruminococcaceae_g__Ruminococcus_s__	0.995763307	2.546279585	0.002997003	-1.550516278
p__Firmicutes_c__Clostridia_o__Clostridiales_NA_NA_NA	1.719289723	0.097669396	0.000999001	1.621620328
p__Firmicutes_c__Erysipelotrichi_o__Erysipelotrichales_f__Erysipelotrichaceae_g__Allobaculum_s__	15.126812	0.103338028	0.002997003	15.02347397
p__Tenericutes_c__Mollicutes_o__RF39_f___g___s__	1.069026606	2.349452393	0.034965035	-1.280425787
p__Verrucomicrobia_c__Verrucomicrobiae_o__Verrucomicrobiales_f__Verrucomicrobiaceae_g__Akkermansia_s__muciniphila	14.16013135	2.809304671	0.035964036	11.35082668

* MRF = mean relative frequency

*DBM = Difference between means

Table S3: Analyses of Composition of Microbiome (ANCOM) between the CD1 mice on protein deficient (PD) and Normal Chow diet (NC). All the Exact Sequence Variants (ESVs) were identified after applying Benjamini-Hochberg (BH) False Discovery Rate (FDR) correction to P-values (< 0.05).

ESVs	CD-1_PD MRF (%)	CD-1_NC MRF (%)	p-values	p-values (corrected)	DBM	95.00% lower CI	95.00% upper CI
p__Bacteroidetes_c__Bacteroidia_o__Bacteroidales_f__Bacteroidaceae_g__Bacteroides_s__	5.961170847	11.79927773	0.033966034	0.033966034	-5.838106885	-10.58238902	-1.296465696
p__Bacteroidetes_c__Bacteroidia_o__Bacteroidales_f__Porphyromonadaceae_g__Parabacteroides_s__	5.218757926	0.680956006	0.000999001	0.000999001	4.537801919	2.395915767	7.03275376
p__Bacteroidetes_c__Bacteroidia_o__Bacteroidales_f__Rikenellaceae_g___s__	8.609144104	24.77429482	0.000999001	0.000999001	-16.16515072	-21.79474372	-9.936120245
p__Firmicutes_c__Erysipelotrichi_o__Erysipelotrichales_f__Erysipelotrichaceae_g__Allobaculum_s__	12.04060811	0.270857565	0.011988012	0.011988012	11.76975055	3.76720245	20.77508601
p__Tenericutes_c__Mollicutes_o__RF39_f___g___s__	0.550671174	3.106752661	0.001998002	0.001998002	-2.556081487	-4.079267334	-1.336232394
p__Verrucomicrobia_c__Verrucomicrobiae_o__Verrucomicrobiales_f__Verrucomicrobiaceae_g__Akkermansia_s__muciniphila	15.30805802	0.142234966	0.015984016	0.015984016	15.16582305	4.320051176	26.37619697

* MRF = mean relative frequency

*DBM = differences between means

Table S4: Analyses of Composition of Microbiome (ANCOM) between the FVB mice on protein deficient (PD) and Normal Chow diet (NC). All the Exact Sequence Variants (ESVs) were identified after applying Benjamini-Hochberg (BH) False Discovery Rate (FDR) correction to P-values (< 0.05).

ESVs	FVB_PD MRF (%)	FVB_NC MRF (%)	p-values (corrected)	DBM	95.00% lower CI	95.00% upper CI
p__Bacteroidetes_c__Bacteroidia_o__Bacteroidales_f__[Odoribacteraceae]_g__Odoribacter_s__	2.179803547	9.054081445	0.000999001	-6.874277898	-8.829845371	-4.732851243
p__Bacteroidetes_c__Bacteroidia_o__Bacteroidales_f__[Paraprevotellaceae]_g__[Prevotella]_s__	3.97616263	0.995719462	0.01998002	2.980443168	1.044897522	4.989784766
p__Bacteroidetes_c__Bacteroidia_o__Bacteroidales_f__Bacteroidaceae_g__Bacteroides_s__	12.97563937	2.875019	0.003996004	10.10062037	4.935434595	14.98570121
p__Bacteroidetes_c__Bacteroidia_o__Bacteroidales_f__Porphyromonadaceae_g__Parabacteroides_s__	4.195427289	0.055279888	0.000999001	4.140147401	1.683107711	6.650745806
p__Bacteroidetes_c__Bacteroidia_o__Bacteroidales_f__Prevotellaceae_g__Prevotella_s__	0.052157205	1.271045616	0.008991009	-1.218888411	-1.826469309	-0.533451963
p__Bacteroidetes_c__Bacteroidia_o__Bacteroidales_f__Rikenellaceae_g___s__	6.675540043	20.99452387	0.000999001	-14.31898383	-17.51306516	-11.08097071
p__Firmicutes_c__Bacilli_o__Lactobacillales_f__Lactobacillaceae_g__Lactobacillus_s__	0.562483008	7.138844722	0.000999001	-6.576361714	-11.29919933	-2.896728606
p__Firmicutes_c__Bacilli_o__Turicibacterales_f__Turicibacteraceae_g__Turicibacter_s__	2.938128919	0.060790304	0.005994006	2.877338616	0.60765151	5.906260189
p__Firmicutes_c__Clostridia_o__Clostridiales_NA_NA_NA	1.310231443	0.005337011	0.007992008	1.304894432	0.024675569	3.483989987
p__Firmicutes_c__Erysipelotrichi_o__Erysipelotrichales_f__Erysipelotrichaceae_g__Allobaculum_s__	2.628916111	0.012711797	0.036963037	2.616204315	0.042832248	6.370409709

* MRF = mean relative frequency

*DBM = differences between means

Table S5: Analyses of Composition of Microbiome (ANCOM) between the NIH-Swiss mice on protein deficient (PD) and Normal Chow diet (NC). All the Exact Sequence Variants (ESVs) were identified after applying Benjamini-Hochberg (BH) False Discovery Rate (FDR) correction to P-values (< 0.05).

ESVs	NIH_PD MRF (%)	NIH_NC MRF (%)	p-values (corrected)	DBM	95.00% lower CI	95.00% upper CI
p__Bacteroidetes_c__Bacteroidia_o__Bacteroidales_f___g___s__	4.596435755	2.606381017	0.003996004	1.990054738	0.775749617	3.350282479
p__Bacteroidetes_c__Bacteroidia_o__Bacteroidales_f__Bacteroidaceae_g__Bacteroides_s__	7.870870359	3.294205303	0.006993007	4.576665056	1.831352104	7.339615824
p__Bacteroidetes_c__Bacteroidia_o__Bacteroidales_f__Porphyromonadaceae_g__Parabacteroides_s__	2.593676576	0.270767954	0.000999001	2.322908622	1.216451802	3.677221329
p__Bacteroidetes_c__Bacteroidia_o__Bacteroidales_f__Prevotellaceae_g__Prevotella_s__	0.195519067	2.005115036	0.000999001	-1.809595969	-2.591999716	-1.052870551
p__Deferribacteres_c__Deferribacteres_o__Deferribacterales_f__Deferribacteraceae_g__Mucispirillum_s__schaedleri	4.882375652	0.363490914	0.00999001	4.518884738	0.963211265	9.082347456
p__Firmicutes_c__Bacilli_o__Lactobacillales_f__Lactobacillaceae_g__Lactobacillus_s__	4.115939722	9.963881723	0.008991009	-5.847942001	-10.07966238	-1.703718843
p__Firmicutes_c__Bacilli_o__Turicibacterales_f__Turicibacteraceae_g__Turicibacter_s__	0.165375546	1.730962078	0.006993007	-1.565586532	-2.558147174	-0.571966646
p__Firmicutes_c__Erysipelotrichi_o__Erysipelotrichales_f__Erysipelotrichaceae_g__Allobaculum_s__	15.54790187	4.272447977	0.03996004	11.27545389	1.471772376	20.47816567

* MRF = mean relative frequency

*DBM = Difference between means

Table S6: Analyses of Composition of Microbiome (ANCOM) between the four mice strains i.e. C57Bl6/J, CD1, GVB, NIH-Swiss on protein deficient (PD). All the Exact Sequence Variants (ESVs) were identified after applying Benjamini-Hochberg (BH) False Discovery Rate (FDR) correction to P-values (< 0.05).

ESVs	C57BL/6_PD MRF (%)	CD-1_PD MRF (%)	FVB_PD MRF (%)	NIH_PD MRF (%)	p-values (corrected)	Effect size
p__Bacteroidetes_c__Bacteroidia_o__Bacteroidales_f__[Odoribacteraceae]_g__Butyricimonas_s__	0	0	0	0.598086696	3.94E-05	0.583111289
p__Bacteroidetes_c__Bacteroidia_o__Bacteroidales_f__[Paraprevotellaceae]_g__[Prevotella]_s__	0	0.000925412	3.97616263	0	2.79E-05	0.57058755
p__Bacteroidetes_c__Bacteroidia_o__Bacteroidales_f__Prevotellaceae_g__Prevotella_s__stercorea	0.000993394	0	0.067703184	0	0.000165119	0.4015227
p__Bacteroidetes_c__Bacteroidia_o__Bacteroidales_f__VC21_Bac22_g___s__	0	0	0	0.065457026	1.97E-05	0.537290454
p__Cyanobacteria_c__4C0d-2_o__YS2_f___g___s__	0	0	1.204473472	0.008689496	2.06E-05	0.581578549
p__Firmicutes_c__Bacilli_o__Lactobacillales_f__Enterococcaceae_g__Enterococcus_s__	0.000895334	0.032617736	0.624418858	0.172839951	5.52E-05	0.419432724
p__Firmicutes_c__Clostridia_o__Clostridiales_f__Clostridiaceae_g___s__	0.096965297	0.006016408	0.008961138	0.009784228	0.000645956	0.473695009
p__Firmicutes_c__Clostridia_o__Clostridiales_f__Clostridiaceae_g__Clostridium_s__	1.379540459	0	0	0.000822639	2.59E-05	0.495650487
p__Firmicutes_c__Clostridia_o__Clostridiales_f__Lachnospiraceae_g__Coprococcus_s__	1.734292665	0.147386783	0.583097855	0.611759009	0.001693053	0.49370888
p__Firmicutes_c__Clostridia_o__Clostridiales_f__Peptococcaceae_g___s__	0.011950043	0.007380854	0.117481056	0.053136193	0.000175482	0.510554261
p__Proteobacteria_c__Deltaproteobacteria_o__Desulfovibrionales_f__Desulfovibrionaceae_g__Bilophila_s__	0	0.460395893	0	0	4.67E-05	0.416917741

* MRF = mean relative frequency

Table S7: Analyses of Composition of Microbiome (ANCOM) between the C57Bl6/J and CD1 mice on protein deficient (PD). All the Exact Sequence Variants (ESVs) were identified after applying Benjamini-Hochberg (BH) False Discovery Rate (FDR) correction to P-values (< 0.05).

ESVs	C57BL/6_PD MRF (%)	CD-1_PD MRF (%)	p-values (corrected)	DBM	95.00% lower CI	95.00% upper CI
p__Bacteroidetes_c__Bacteroidia_o__Bacteroidales_f__Porphyromonadaceae_g__Parabacteroides_s__	0.454178651	5.218757926	0.002997003	-4.764579274	-7.119851549	-2.470188952
p__Bacteroidetes_c__Bacteroidia_o__Bacteroidales_f__Rikenellaceae_g___s__	5.092540517	8.609144104	0.017982018	-3.516603587	-5.806619098	-0.808456873
p__Firmicutes_c__Clostridia_o__Clostridiales_f__Clostridiaceae_g__Clostridium_s__	1.379540459	0	0.000999001	1.379540459	0.701317811	2.124116226
p__Firmicutes_c__Clostridia_o__Clostridiales_f__Lachnospiraceae_g__Coprococcus_s__	1.734292665	0.147386783	0.000999001	1.586905882	0.960235061	2.273205644
p__Firmicutes_c__Clostridia_o__Clostridiales_f__Ruminococcaceae_g___s__	2.978643617	1.768880004	0.018981019	1.209763613	0.306967833	2.036173214
p__Firmicutes_c__Clostridia_o__Clostridiales_f__Ruminococcaceae_g__Oscillospira_s__	2.103149904	1.006067273	0.002997003	1.097082631	0.496989621	1.710651552

* MRF = mean relative frequency

*DBM = Difference between means

Table S8: Rodent Diet Composition

	NC	PD
Macronutrients	%	%
Protein	19.1	6.1
Carbohydrate	44.3	75.6
Fiber	4.6	4.6
Fat	5.8	5.5

From Teklad Custom Diet (Envigo):
Normal Chow (NC): LM-485 (7012)
Protein deficient diet (PD): TD.90016

Table S9: Overall microbial community profiles in the 4 strain of mice

sOTUs	Mean rel. freq (%)							
	C57BL6PD	C57BL6-NC	CD1-PD	CD1-NC	FVB-PD	FVB-NC	NH-PD	NIH-NC
p__Actinobacteria_c__Actinobacteria_o__Actinomycetales_f__Corynebacteriaceae_g__Corynebacterium_s__	0	0.013777602	0	0.067352988	0.495013613	0.006291682	0.266863701	0.26080287
p__Actinobacteria_c__Actinobacteria_o__Actinomycetales_f__Micrococcaceae_NA_NA	0	0	0.007738032	0.001631348	0	0	0	0
p__Actinobacteria_c__Actinobacteria_o__Bifidobacteriales_f__Bifidobacteriaceae_g__Bifidobacterium_s__pseudolongum	0.921718224	0.505991677	3.367542953	2.081861431	0.042440379	0.030567831	3.272460053	4.396623639
p__Actinobacteria_c__Coriobacteriia_o__Coriobacteriales_f__Coriobacteriaceae_g___s__	0.885752051	0.324172817	0.001028235	0.03866524	0.012243897	0.005966377	0.004153393	0
p__Actinobacteria_c__Coriobacteriia_o__Coriobacteriales_f__Coriobacteriaceae_g__Adlercreutzia_s__	0.027415943	0.045377391	0.165288825	0.124984983	0.06289623	0.03624026	0.051780981	0.066639379
p__Bacteroidetes_c__Bacteroidia_o__Bacteroidales_f__[Odoribacteraceae]_g__Butyricimonas_s__	0	0	0	0.001251322	0	0	0.614401744	0.053010363
p__Bacteroidetes_c__Bacteroidia_o__Bacteroidales_f__[Odoribacteraceae]_g__Odoribacter_s__	0.176592919	0	0.001518741	0.010721198	1.567304694	9.187697993	1.671804029	2.369290079
p__Bacteroidetes_c__Bacteroidia_o__Bacteroidales_f__[Paraprevotellaceae]_g__[Prevotella]_s__	0	0	0.001028235	0	3.513471291	1.069619173	0	0.002257744
p__Bacteroidetes_c__Bacteroidia_o__Bacteroidales_f___g___s__	0.826391132	0	1.125643816	0.058684453	3.004722028	5.006375817	4.370949525	2.831867247
p__Bacteroidetes_c__Bacteroidia_o__Bacteroidales_f__Bacteroidaceae_g__Bacteroides_s__	6.614145077	2.775805537	6.024272226	11.93812675	14.21901807	2.879747145	7.310922063	3.8541536
p__Bacteroidetes_c__Bacteroidia_o__Bacteroidales_f__Bacteroidaceae_g__Bacteroides_s__caccae	0	0	0.306136797	0	0	0	0	0
p__Bacteroidetes_c__Bacteroidia_o__Bacteroidales_f__Bacteroidaceae_g__Bacteroides_s__uniformis	0	0	0.472479908	0.273044682	0	0	0.073727503	0
p__Bacteroidetes_c__Bacteroidia_o__Bacteroidales_f__Porphyromonadaceae_g__Parabacteroides_s__	0.504642946	0.000574515	5.278681605	0.667556305	3.380869335	0.060111852	2.777358946	0.087085584
p__Bacteroidetes_c__Bacteroidia_o__Bacteroidales_f__Porphyromonadaceae_g__Parabacteroides_s__distasonis	0	0.001270245	0	0	0.162631185	0.006016224	0.267023382	0.044475038
p__Bacteroidetes_c__Bacteroidia_o__Bacteroidales_f__Prevotellaceae_g__Prevotella_s__	0.008191216	0.320022262	0	0.14607619	0.035627012	1.217967989	0.084091592	2.116542512
p__Bacteroidetes_c__Bacteroidia_o__Bacteroidales_f__Prevotellaceae_g__Prevotella_s__stercorea	0.001103771	0	0	0	0.076166082	0.479029917	0	0
p__Bacteroidetes_c__Bacteroidia_o__Bacteroidales_f__Rikenellaceae_g___s__	5.418059524	10.21602395	8.682995542	23.78253985	6.86877781	20.45678128	3.771034901	11.58997543
p__Bacteroidetes_c__Bacteroidia_o__Bacteroidales_f__Rikenellaceae_g__AF12_s__	0.190156605	0	0	0.003385641	0.450195244	0.716080649	0.489861449	0.502110633
p__Bacteroidetes_c__Bacteroidia_o__Bacteroidales_f__S24-7_g___s__	19.48468664	48.04930871	19.83997043	21.8800574	30.15342456	28.17354865	17.21220596	29.07339548
p__Bacteroidetes_c__Bacteroidia_o__Bacteroidales_f__VC21_Bac22_g___s__	0	0	0	0	0	0	0.063006947	0.023207664
p__Cyanobacteria_c__4C0d-2_o__YS2_f___g___s__	0	0	0	0	1.060972531	0.40959608	0.008689496	0.040046536
p__Cyanobacteria_c__Chloroplast_o__Streptophyta_f___g___s__	0	0	0	0.003568115	0	0.002164127	0	0.002368151
p__Deferribacteres_c__Deferribacteres_o__Deferribacterales_f__Deferribacteraceae_g__Mucispirillum_s__schaedleri	1.042016887	0.000648298	2.660658547	3.35317471	2.144227305	0.822908683	4.743449026	0.50241754
p__Firmicutes_c__Bacilli_o__Bacillales_f__Staphylococcaceae_g__Jeotgalicoccus_s__	0	0	0	0.003494582	0	0	0.001757901	0.002605015
p__Firmicutes_c__Bacilli_o__Bacillales_f__Staphylococcaceae_g__Staphylococcus_s__	0.00188359	0	0.004207724	0.002574391	0	0.001634094	0.001780444	0
p__Firmicutes_c__Bacilli_o__Bacillales_f__Staphylococcaceae_g__Staphylococcus_s__succinus	0.002918409	0.20887	0.021454738	0.122680582	0.605263907	0.120651376	0.149798758	0.526445946
p__Firmicutes_c__Bacilli_o__Lactobacillales_f__Aerococcaceae_g__Aerococcus_s__	0	0	0.013785498	0.060437359	0.002305989	0.002359381	0.034885619	0.002488813
p__Firmicutes_c__Bacilli_o__Lactobacillales_f__Enterococcaceae_g__Enterococcus_s__	0.000994815	0.000635122	0.036241929	0.001193336	0.610970321	0.07627347	0.175673011	0.045181044
p__Firmicutes_c__Bacilli_o__Lactobacillales_f__Enterococcaceae_g__Vagococcus_s__	0	0	0	0	0.018662108	0	0.00230716	0
p__Firmicutes_c__Bacilli_o__Lactobacillales_f__Lactobacillaceae_g__Lactobacillus_s__	1.32420948	0.739339881	4.162392826	9.309812194	0.486112561	7.823398029	3.466059843	10.6137616
p__Firmicutes_c__Bacilli_o__Lactobacillales_f__Lactobacillaceae_g__Lactobacillus_s__reuteri	0.001638243	0.001391401	0.002110372	0.638675423	0	0.409053955	0	0.472782304
p__Firmicutes_c__Bacilli_o__Lactobacillales_f__Streptococcaceae_g__Lactococcus_s__	0.226716616	0	0.364510489	0.010721198	0.36280839	0.004608012	0.123829436	0.001354646
p__Firmicutes_c__Bacilli_o__Lactobacillales_f__Streptococcaceae_g__Streptococcus_s__	0	0	0.014008422	0.003262696	0.015575843	0	0.001290739	0
p__Firmicutes_c__Bacilli_o__Turicibacterales_f__Turicibacteraceae_g__Turicibacter_s__	1.150354199	3.702252816	0.66579066	0.053096374	3.2152911	0.068389092	0.134845295	1.761492328
p__Firmicutes_c__Clostridia_o__Clostridiales_f__[Mogibacteriaceae]_g___s__	0.002984446	0.023314102	0.030998266	0.059045969	0.042549026	0.023324496	0.039787298	0.060217639
p__Firmicutes_c__Clostridia_o__Clostridiales_f___g___s__	9.565945255	11.56573548	9.247297507	11.46458303	9.361441787	9.55347265	8.137150681	11.97177046
p__Firmicutes_c__Clostridia_o__Clostridiales_f__Christensenellaceae_g___s__	0.036733726	0.026140508	0.037924475	0.040180377	0.034639709	0.014245897	0.029998001	0.028572587
p__Firmicutes_c__Clostridia_o__Clostridiales_f__Christensenellaceae_g__Christensenella_s__	0	0	0	0	0	0	0	0.010424532
p__Firmicutes_c__Clostridia_o__Clostridiales_f__Clostridiaceae_g___s__	0.100897401	0.038669335	0.006684897	0.094776663	0.01008128	0.010597556	0.007946668	0.014262986
p__Firmicutes_c__Clostridia_o__Clostridiales_f__Clostridiaceae_g__Candidatus Arthromitus_s__	0	0.016696814	0.013242413	0.879737049	0	0.121021463	0.002258286	0.044908766
p__Firmicutes_c__Clostridia_o__Clostridiales_f__Clostridiaceae_g__Clostridium_s__	1.501179327	0.899128453	0	0.034455739	0	0.005240973	0.000822639	0.416378054
p__Firmicutes_c__Clostridia_o__Clostridiales_f__Dehalobacteriaceae_g__Dehalobacterium_s__	0.063468619	0.103376291	0.105035468	0.08658945	0.094589111	0.053614575	0.089966755	0.101351687
p__Firmicutes_c__Clostridia_o__Clostridiales_f__Eubacteriaceae_g__Anaerofustis_s__	0	0.001270245	0	0.016385788	0	0	0	0.002914968

Table S9 cont

p__Firmicutes_c__Clostridia_o__Clostridiales_f__Lachnospiraceae_g__[Ruminococcus]_s__gnavus	0.904295862	0.42517821	0.28840447	0.133493162	0.626105427	0.14979878	0.269752834	0.371009317
p__Firmicutes_c__Clostridia_o__Clostridiales_f__Lachnospiraceae_g___s__	4.931873455	4.041115447	3.587641521	3.267343769	4.024744667	3.144055626	2.263688731	5.526069613
p__Firmicutes_c__Clostridia_o__Clostridiales_f__Lachnospiraceae_g__Anaerostipes_s__	0.028020551	0.489065717	0.003822637	0.034886074	0	0.003358486	0	0.066417607
p__Firmicutes_c__Clostridia_o__Clostridiales_f__Lachnospiraceae_g__Blautia_s__	0	0.001574431	0.004657244	0.002447022	0.002620619	0	0	0.001302507
p__Firmicutes_c__Clostridia_o__Clostridiales_f__Lachnospiraceae_g__Coprococcus_s__	1.504509639	0.221109571	0.116763018	0.141546679	0.542831309	0.11134934	0.609736508	0.178986905
p__Firmicutes_c__Clostridia_o__Clostridiales_f__Lachnospiraceae_g__Dorea_s__	0.009625746	0.017852561	0.04680431	0.025747165	0.059170693	0.015107709	0.007770178	0.019360479
p__Firmicutes_c__Clostridia_o__Clostridiales_f__Lachnospiraceae_g__Epulopiscium_s__	0	0	0	0	0.072111135	0.000723045	0.017915463	0
p__Firmicutes_c__Clostridia_o__Clostridiales_f__Lachnospiraceae_g__Roseburia_NA	0	0	0	0	0	0	0.010183299	0
p__Firmicutes_c__Clostridia_o__Clostridiales_f__Lachnospiraceae_g__Roseburia_s__	0.001655657	0.024769768	0	0.026735988	0.010832119	0.014342561	0.019966401	0.004481308
p__Firmicutes_c__Clostridia_o__Clostridiales_f__Lachnospiraceae_NA_NA	0.016325825	0.000618353	0.119256211	0.03850978	0.348080859	0.045232802	0.170454848	0.067241231
p__Firmicutes_c__Clostridia_o__Clostridiales_f__Peptococcaceae_g___s__	0.013277826	0.009134025	0.008200949	0.001128547	0.125181387	0.01838288	0.048848553	0.026152058
p__Firmicutes_c__Clostridia_o__Clostridiales_f__Peptococcaceae_g__rc4-4_s__	0	0.001471454	0.044922225	0.407664339	0	0	0.00161773	0.001475361
p__Firmicutes_c__Clostridia_o__Clostridiales_f__Ruminococcaceae_g___s__	2.88626658	3.424827019	1.797984465	1.254882728	1.509459999	1.43822216	1.340124081	1.879461335
p__Firmicutes_c__Clostridia_o__Clostridiales_f__Ruminococcaceae_g__Anaerotruncus_s__	0.004227598	0	0.003598824	0	0.008330805	0	0.005632164	0
p__Firmicutes_c__Clostridia_o__Clostridiales_f__Ruminococcaceae_g__Oscillospira_s__	1.98790055	1.209412187	1.048821167	1.27310723	1.276878414	0.749885773	1.166653057	1.623136213
p__Firmicutes_c__Clostridia_o__Clostridiales_f__Ruminococcaceae_g__Ruminococcus_NA	0	0	0	0	0.025132856	0.664896275	0	0.045760224
p__Firmicutes_c__Clostridia_o__Clostridiales_f__Ruminococcaceae_g__Ruminococcus_s__	0.96358074	2.546279585	0.77133755	1.1026525	0.742637476	0.785880575	0.450691914	0.985185539
p__Firmicutes_c__Clostridia_o__Clostridiales_f__Ruminococcaceae_g__Ruminococcus_s__flavefaciens	0.002730405	0	0	0.002821368	0	0	0.445415558	0.184081093
p__Firmicutes_c__Clostridia_o__Clostridiales_f__Ruminococcaceae_NA_NA	0.001453193	0	0	0.001631348	0.008105748	0.061938623	0.004448758	0.094681721
p__Firmicutes_c__Clostridia_o__Clostridiales_f__Veillonellaceae_g__Veillonella_s__dispar	0.001103771	0	0.001406915	0.002861349	0	0	0.002397485	0.000903098
p__Firmicutes_c__Clostridia_o__Clostridiales_NA_NA_NA	1.782037843	0.097669396	0.882028945	0.042193383	1.474010373	0.006004137	0.181399202	0.40611353
p__Firmicutes_c__Erysipelotrichi_o__Erysipelotrichales_f__Erysipelotrichaceae_g___s__	0.972083581	0.337280746	0.269794993	0.676093083	0.054721426	0.081591172	0.069297669	0.183250213
p__Firmicutes_c__Erysipelotrichi_o__Erysipelotrichales_f__Erysipelotrichaceae_g__Allobaculum_s__	15.23138526	0.103338028	12.75570249	0.30095285	2.954736705	0.014300771	18.62396655	1.196383296
p__Firmicutes_c__Erysipelotrichi_o__Erysipelotrichales_f__Erysipelotrichaceae_g__Coprobacillus_s__	0.080710933	0.005764974	0	0.007299653	0.000967193	0	0	0.002075119
p__Firmicutes_c__Erysipelotrichi_o__Erysipelotrichales_f__Erysipelotrichaceae_NA_NA	0	0.003400655	0	0.015406603	0.012189176	0.012287071	0.003394433	0.005640226
p__Proteobacteria_c__Alphaproteobacteria_o___f___g___s__	0	0	0	0	0.002636436	0.006683894	0	0
p__Proteobacteria_c__Alphaproteobacteria_o__RF32_f___g___s__	0.004606312	0.019053668	0.023831064	0.001128547	0.790445153	0.212088548	0.39291771	0.150834603
p__Proteobacteria_c__Alphaproteobacteria_o__Rickettsiales_f__mitochondria_NA_NA	0	0	0	0.001703026	0	0.008226166	0	0.002075119
p__Proteobacteria_c__Betaproteobacteria_o__Burkholderiales_f__Alcaligenaceae_g__Achromobacter_s__	0	0	0	0	0.023504101	0.012221464	0	0
p__Proteobacteria_c__Betaproteobacteria_o__Burkholderiales_f__Alcaligenaceae_g__Sutterella_s__	1.362927192	1.224363878	0.085997216	0.085358147	0	0	0	0
p__Proteobacteria_c__Betaproteobacteria_o__Burkholderiales_f__Oxalobacteraceae_NA_NA	0	0	0	0.01010026	0	0	0	0
p__Proteobacteria_c__Betaproteobacteria_o__Methylophilales_f___g___s__	0.009509681	0	0	0	0.253265044	0.002728776	1.561159882	0.016925208
p__Proteobacteria_c__Betaproteobacteria_o__Rhodocyclales_f__Rhodocyclaceae_g__Dok59_s__	0	0	0	0	0.004306333	0.009913493	0.02312655	0.148144712
p__Proteobacteria_c__Deltaproteobacteria_o__Desulfovibrionales_f__Desulfovibrionaceae_g___s__	0.304272869	0.349859818	0.059793543	0	0.524147414	0.043222917	0.451466289	0.058980979
p__Proteobacteria_c__Deltaproteobacteria_o__Desulfovibrionales_f__Desulfovibrionaceae_g__Bilophila_s__	0	0	0.457207157	0.00998057	0	0	0	0
p__Proteobacteria_c__Deltaproteobacteria_o__Desulfovibrionales_f__Desulfovibrionaceae_g__Desulfovibrio_s__	0.406543763	0.129178377	0.369644723	0.087363994	0.486382304	0.239052354	0.566236605	0.463617886
p__Proteobacteria_c__Gammaproteobacteria_o__Enterobacteriales_f__Enterobacteriaceae_g___s__	0.016631617	0.011891327	0.573262662	0.079137925	0.302408635	0.198551721	0.403458476	0.102030129
p__Proteobacteria_c__Gammaproteobacteria_o__Enterobacteriales_f__Enterobacteriaceae_g__Proteus_s__	0	0	0.01410098	0.002821368	0	0	0	0
p__Proteobacteria_c__Gammaproteobacteria_o__Pasteurellales_f__Pasteurellaceae_g__Haemophilus_s__parainfluenzae	0.001103771	0	0	0.001505979	0.001318218	0	0.002035937	0
p__Proteobacteria_c__Gammaproteobacteria_o__Pseudomonadales_f__Moraxellaceae_g__Acinetobacter_s__venetianus	0.001092162	0	0	0	0.017806771	0.001572921	0.020635691	0
p__Tenericutes_c__Mollicutes_o__Anaeroplasmatales_f__Anaeroplasmataceae_g__Anaeroplasma_s__	0.356375303	0.55469394	0.053563503	0.001128547	0.393870619	0.420908684	1.018779174	0.138065335
p__Tenericutes_c__Mollicutes_o__RF39_f___g___s__	1.174978932	2.349452393	0.5736693	3.117828556	1.11318831	2.511248003	0.449010281	1.888943964
p__TM7_c__TM7-3_o__CW040_f__F16_g___s__	0	0.010083915	0.036751632	0.193940334	0.030101949	0.044441779	0.004813242	0.031578478
p__Verrucomicrobia_c__Verrucomicrobiae_o__Verrucomicrobiales_f__Verrucomicrobiaceae_g__Akkermansia_s__muciniphila	14.95522257	2.809304671	13.31063508	0.15471979	0.011773016	0.01950953	9.889472665	0.188600827
	0.002873761	0.002462448	0.029725635	0.179493529	0.055704906	0.097675215	0.04958886	0.047851909

Fig. S1

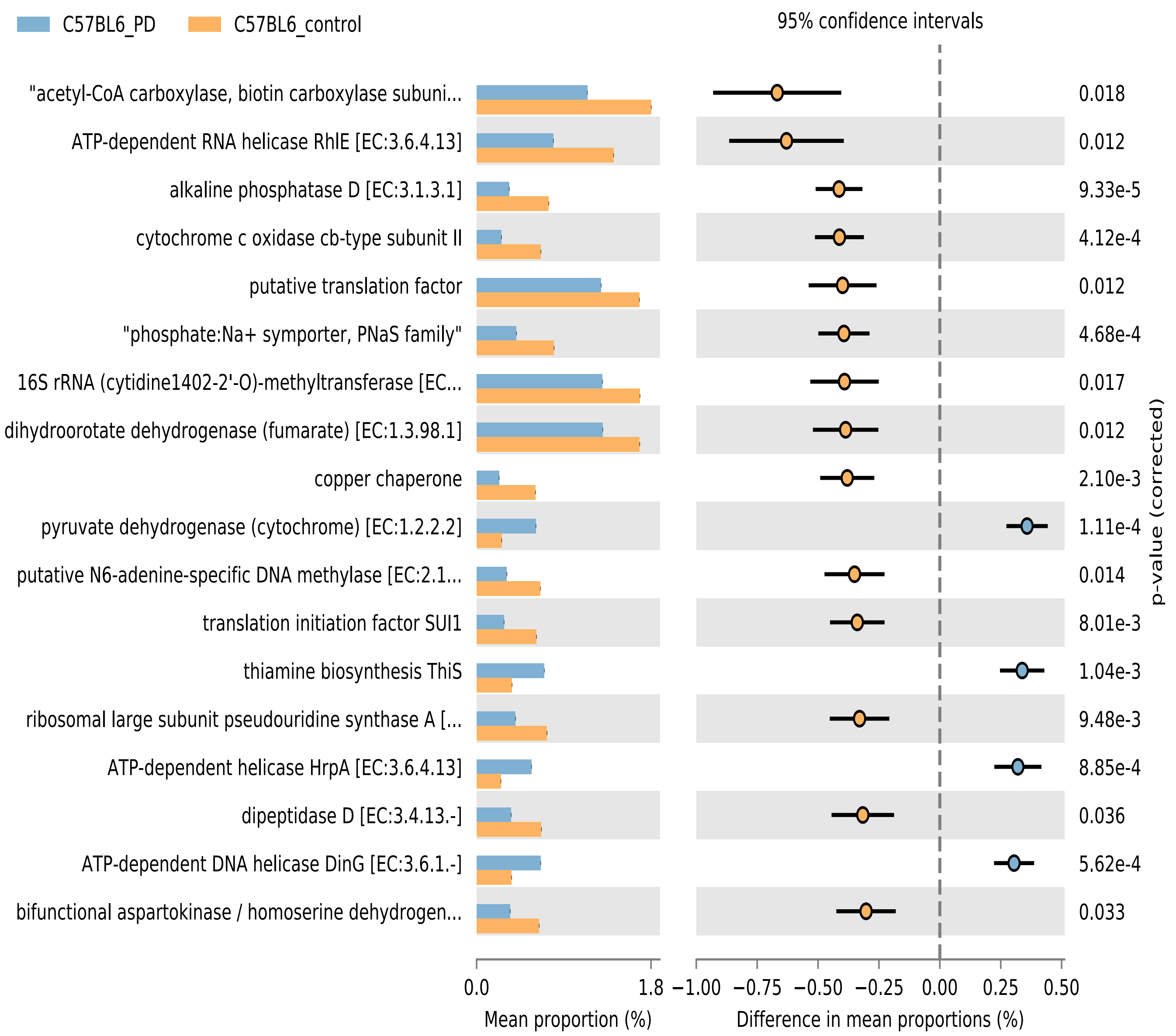

C57BL6_PD
C57BL6_control
95% confidence intervals
"acetyl-CoA carboxylase, biotin carboxylase subuni...
ATP-dependent RNA helicase RhlE [EC:3.6.4.13]
alkaline phosphatase D [EC:3.1.3.1]
cytochrome c oxidase cb-type subunit II
putative translation factor
"phosphate:Na+ symporter, PNaS family"
16S rRNA (cytidine1402-2'-O)-methyltransferase [EC...
dihydroorotate dehydrogenase (fumarate) [EC:1.3.98.1]
copper chaperone
pyruvate dehydrogenase (cytochrome) [EC:1.2.2.2]
putative N6-adenine-specific DNA methylase [EC:2.1...
translation initiation factor SUI1
thiamine biosynthesis ThiS
ribosomal large subunit pseudouridine synthase A [...
ATP-dependent helicase HrpA [EC:3.6.4.13]
dipeptidase D [EC:3.4.13.-]
ATP-dependent DNA helicase DinG [EC:3.6.1.-]
bifunctional aspartokinase / homoserine dehydrogen...
0.018
0.012
9.33e-5
4.12e-4
0.012
4.68e-4
0.017
0.012
2.10e-3
1.11e-4
0.014
8.01e-3
1.04e-3
9.48e-3
8.85e-4
0.036
5.62e-4
0.033
p-value (corrected)
0.0
1.8
Mean proportion (%)
−1.00
−0.75
−0.50
−0.25
0.00
0.25
0.50
Difference in mean proportions (%)

Fig. S2

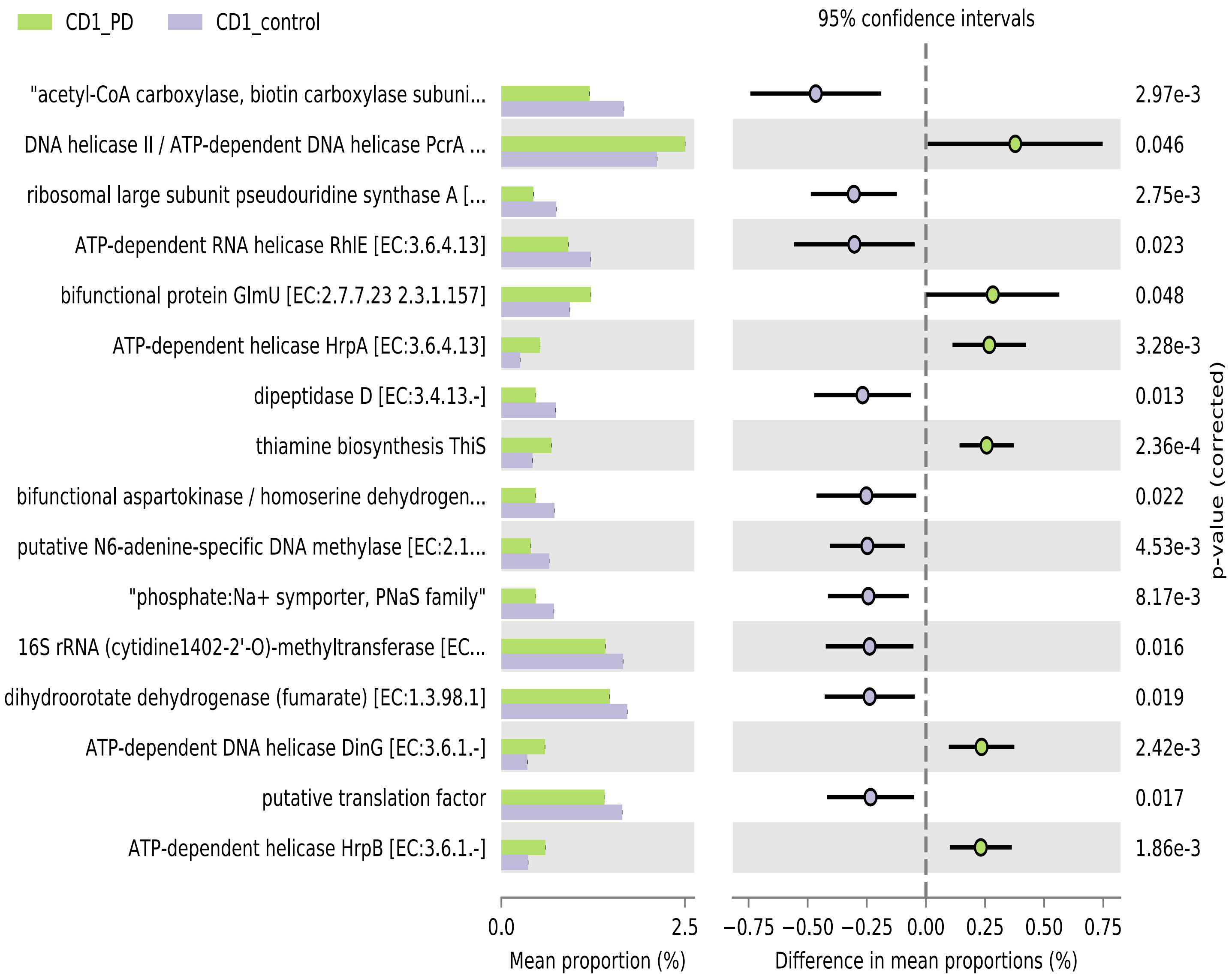

CD1_PD
CD1_control
95% confidence intervals
"acetyl-CoA carboxylase, biotin carboxylase subuni...
DNA helicase II / ATP-dependent DNA helicase PcrA ...
ribosomal large subunit pseudouridine synthase A [...
ATP-dependent RNA helicase RhlE [EC:3.6.4.13]
bifunctional protein GlmU [EC:2.7.7.23 2.3.1.157]
ATP-dependent helicase HrpA [EC:3.6.4.13]
dipeptidase D [EC:3.4.13.-]
thiamine biosynthesis ThiS
bifunctional aspartokinase / homoserine dehydrogen...
putative N6-adenine-specific DNA methylase [EC:2.1...
"phosphate:Na+ symporter, PNaS family"
16S rRNA (cytidine1402-2'-O)-methyltransferase [EC...
dihydroorotate dehydrogenase (fumarate) [EC:1.3.98.1]
ATP-dependent DNA helicase DinG [EC:3.6.1.-]
putative translation factor
ATP-dependent helicase HrpB [EC:3.6.1.-]
2.97e-3
0.046
2.75e-3
0.023
0.048
3.28e-3
0.013
2.36e-4
0.022
4.53e-3
8.17e-3
0.016
0.019
2.42e-3
0.017
1.86e-3
p-value (corrected)
0.0
2.5
Mean proportion (%)
−0.75 −0.50 −0.25 0.00 0.25 0.50 0.75
Difference in mean proportions (%)

Fig. S3

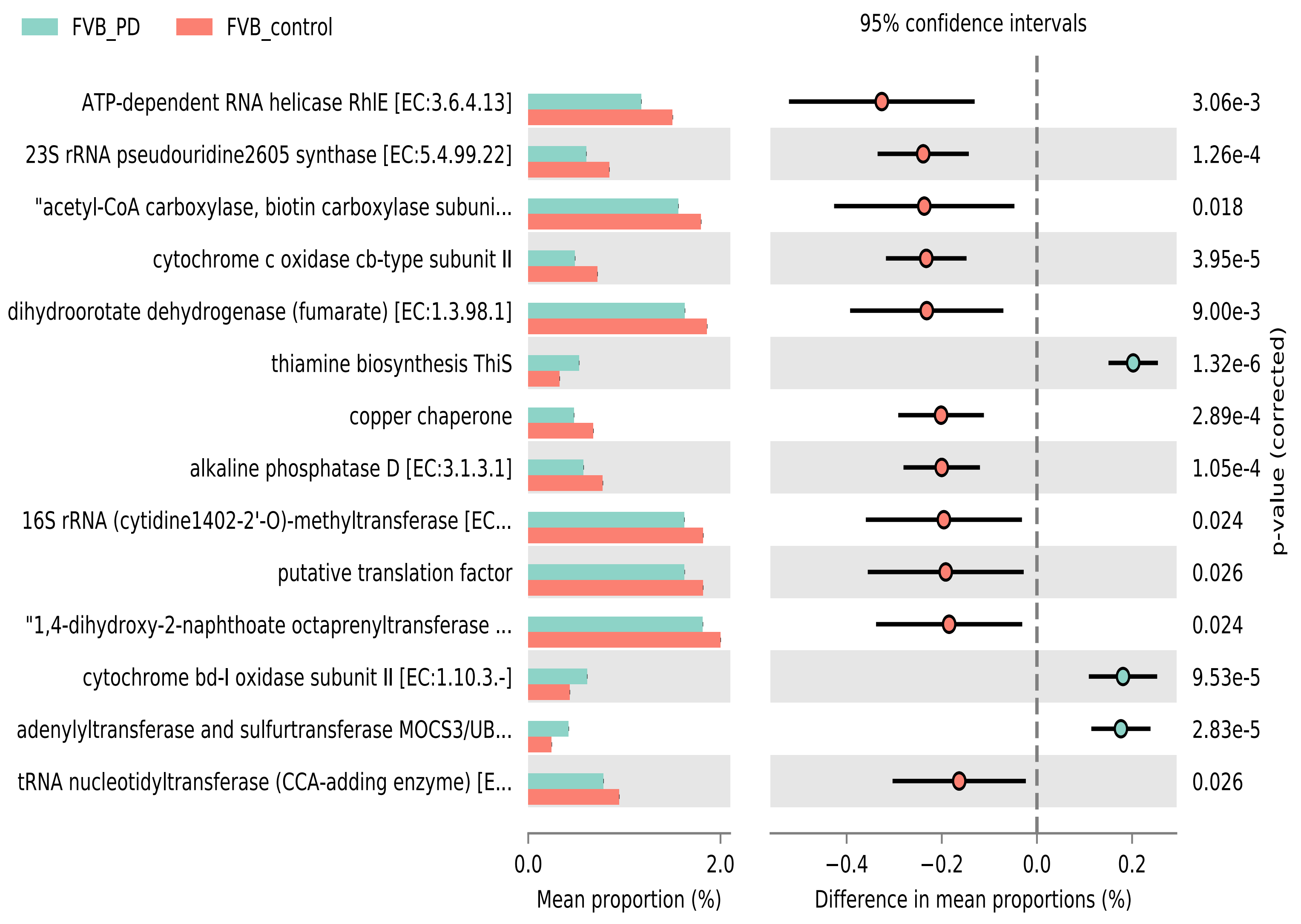

FVB_PD
FVB_control
95% confidence intervals
ATP-dependent RNA helicase RhlE [EC:3.6.4.13]
23S rRNA pseudouridine2605 synthase [EC:5.4.99.22]
"acetyl-CoA carboxylase, biotin carboxylase subuni...
cytochrome c oxidase cb-type subunit II
dihydroorotate dehydrogenase (fumarate) [EC:1.3.98.1]
thiamine biosynthesis ThiS
copper chaperone
alkaline phosphatase D [EC:3.1.3.1]
16S rRNA (cytidine1402-2'-O)-methyltransferase [EC...
putative translation factor
"1,4-dihydroxy-2-naphthoate octaprenyltransferase ...
cytochrome bd-I oxidase subunit II [EC:1.10.3.-]
adenylyltransferase and sulfurtransferase MOCS3/UB...
tRNA nucleotidyltransferase (CCA-adding enzyme) [E...
3.06e-3
1.26e-4
0.018
3.95e-5
9.00e-3
1.32e-6
2.89e-4
1.05e-4
0.024
0.026
0.024
9.53e-5
2.83e-5
0.026
p-value (corrected)
0.0
2.0
Mean proportion (%)
−0.4
−0.2
0.0
0.2
Difference in mean proportions (%)

Fig. S4

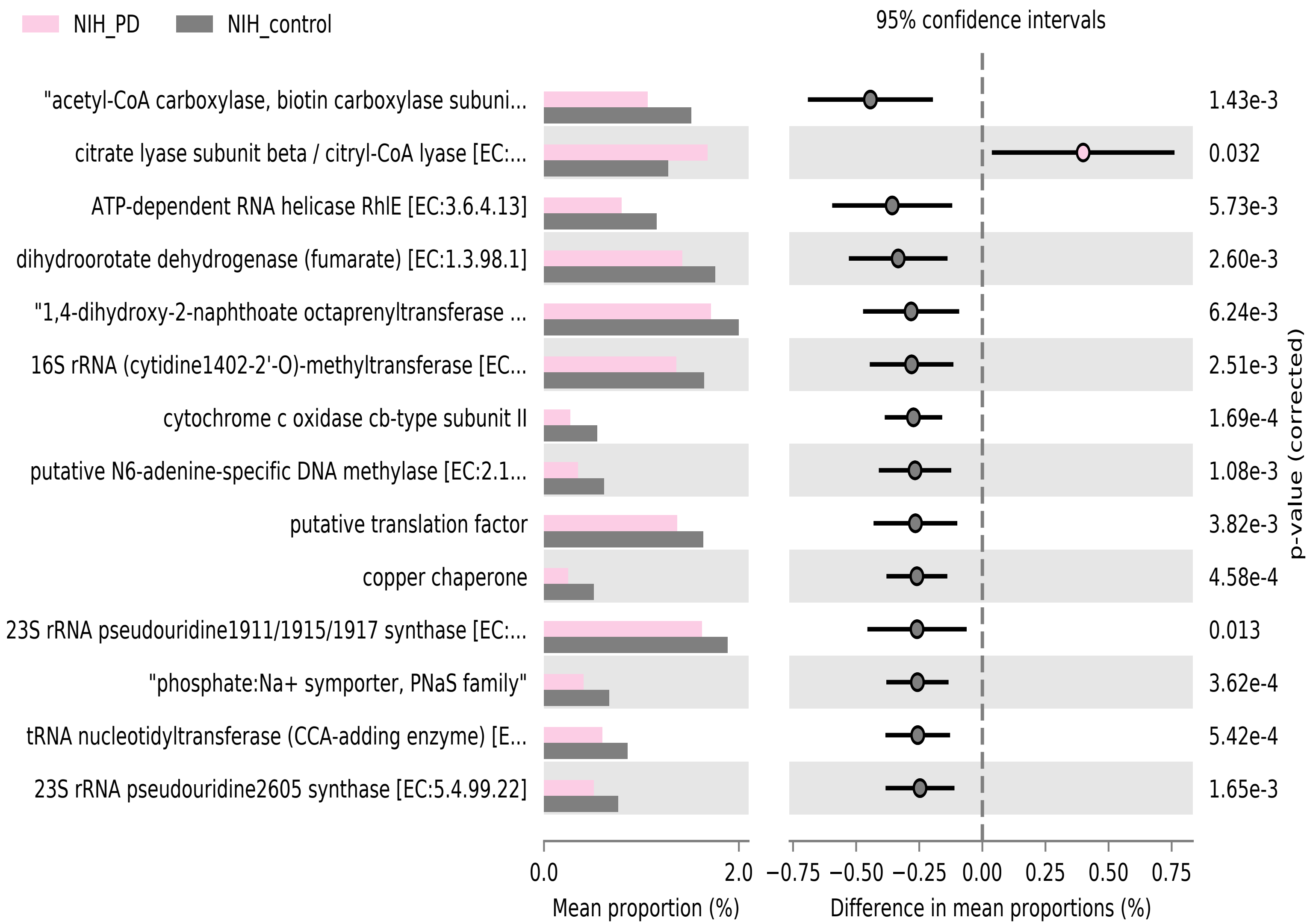
NIH_PD
NIH_control
95% confidence intervals
"acetyl-CoA carboxylase, biotin carboxylase subuni...
citrate lyase subunit beta / citryl-CoA lyase [EC:...
ATP-dependent RNA helicase RhlE [EC:3.6.4.13]
dihydroorotate dehydrogenase (fumarate) [EC:1.3.98.1]
"1,4-dihydroxy-2-naphthoate octaprenyltransferase ...
16S rRNA (cytidine1402-2'-O)-methyltransferase [EC...
cytochrome c oxidase cb-type subunit II
putative N6-adenine-specific DNA methylase [EC:2.1...
putative translation factor
copper chaperone
23S rRNA pseudouridine1911/1915/1917 synthase [EC:...
"phosphate:Na+ symporter, PNaS family"
tRNA nucleotidyltransferase (CCA-adding enzyme) [E...
23S rRNA pseudouridine2605 synthase [EC:5.4.99.22]
1.43e-3
0.032
5.73e-3
2.60e-3
6.24e-3
2.51e-3
1.69e-4
1.08e-3
3.82e-3
4.58e-4
0.013
3.62e-4
5.42e-4
1.65e-3
p-value (corrected)
0.0
2.0
Mean proportion (%)
−0.75
−0.50
−0.25
0.00
0.25
0.50
0.75
Difference in mean proportions (%)

Fig. S5

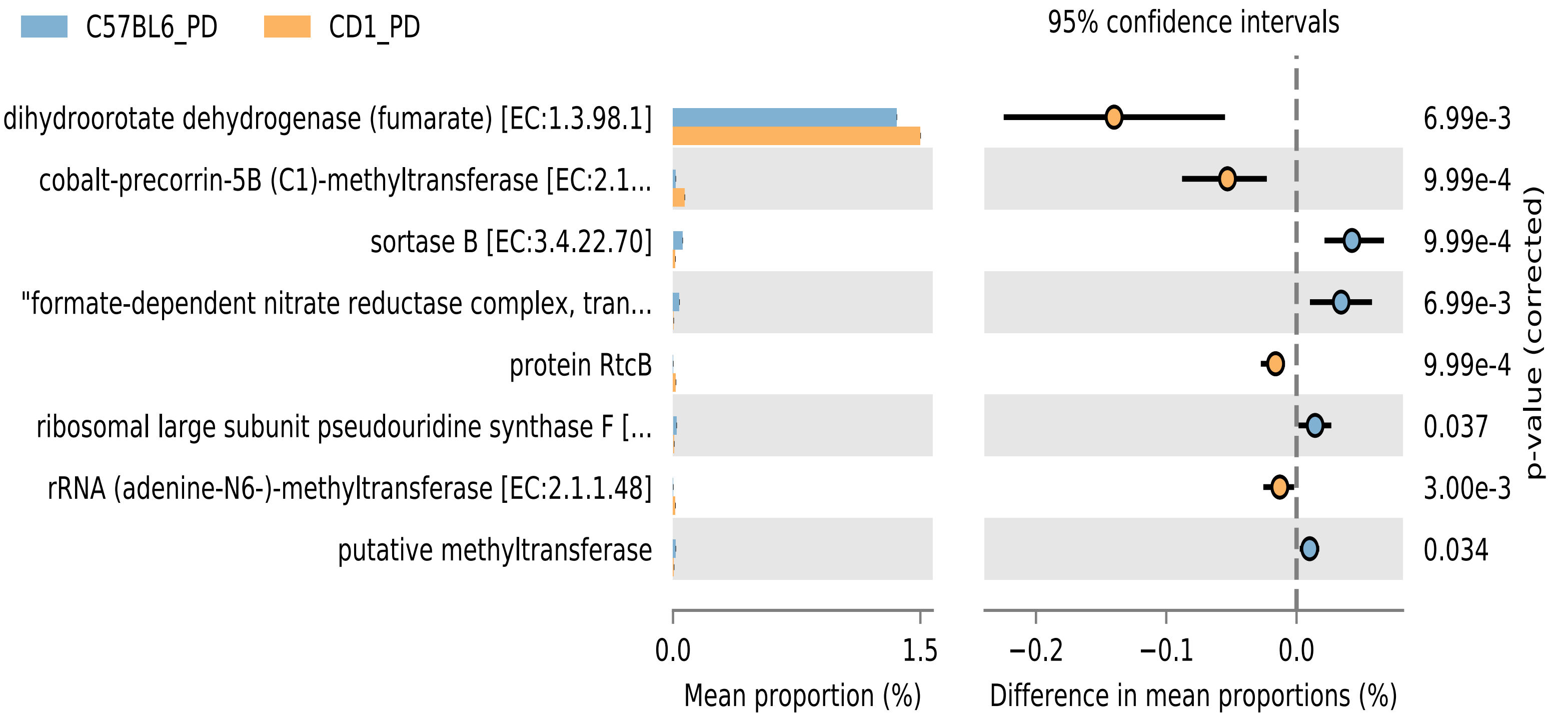

C57BL6_PD
CD1_PD
95% confidence intervals
dihydroorotate dehydrogenase (fumarate) [EC:1.3.98.1]
cobalt-precorrin-5B (C1)-methyltransferase [EC:2.1...
sortase B [EC:3.4.22.70]
"formate-dependent nitrate reductase complex, tran...
protein RtcB
ribosomal large subunit pseudouridine synthase F [...
rRNA (adenine-N6-)-methyltransferase [EC:2.1.1.48]
putative methyltransferase
6.99e-3
9.99e-4
9.99e-4
6.99e-3
9.99e-4
0.037
3.00e-3
0.034
p-value (corrected)
0.0
1.5
Mean proportion (%)
−0.2
−0.1
0.0
Difference in mean proportions (%)

Fig. S6

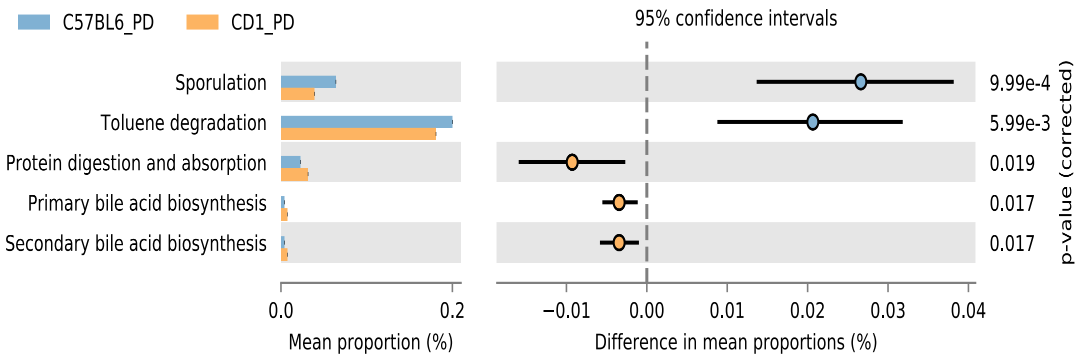
C57BL6_PD
CD1_PD
95% confidence intervals
Sporulation
Toluene degradation
Protein digestion and absorption
Primary bile acid biosynthesis
Secondary bile acid biosynthesis
9.99e-4
5.99e-3
0.019
0.017
0.017
p-value (corrected)
0.0
0.2
Mean proportion (%)
−0.01
0.00
0.01
0.02
0.03
0.04
Difference in mean proportions (%)
